# In Situ Decarboxylation-Pressurized Hot Water Extraction for Selective Extraction of Cannabinoids from *Cannabis sativa.* Chemometric Approach

**DOI:** 10.3390/molecules26113343

**Published:** 2021-06-02

**Authors:** Yannick Nuapia, Kgomotso Maraba, Hlanganani Tutu, Luke Chimuka, Ewa Cukrowska

**Affiliations:** 1 Molecular Sciences Institute, School of Chemistry, University of Witwatersrand, Private Bag X3, Johannesburg 2050, South Africa; Yannick.Nuapia@wits.ac.za (Y.N.); 1687672@students.wits.ac.za (K.M.); hlanganani.tutu@wits.ac.za (H.T.); luke.chimuka@wits.ac.za (L.C.); 2 School of Animal, Plant and Environmental Sciences, University of the Witwatersrand, Johannesburg 2050, South Africa

**Keywords:** decarboxylation, cannabinoid compounds, green extraction

## Abstract

Isolation of the therapeutic cannabinoid compounds from *Cannabis Sativa L.* (*C. Sativa*) is important for the development of cannabis-based pharmaceuticals for cancer treatment, among other ailments. The main pharmacological cannabinoids are THC and CBD. However, THC also induces undesirable psychoactive effects. The decarboxylation process converts the naturally occurring acidic forms of cannabinoids, such as cannabidiolic acid (CBDA) and tetrahydrocannabinolic acid (THCA), to their more active neutral forms, known as cannabidiol (CBD) and tetrahydrocannabinol (THC). The purpose of this study was to selectively extract cannabinoids using a novel in situ decarboxylation pressurized hot water extraction (PHWE) system. The decarboxylation step was evaluated at different temperature (80–150 °C) and time (5–60 min) settings to obtain the optimal conditions for the decarboxylation-PHWE system using response surface methodology (RSM). The system was optimized to produce cannabis extracts with high CBD content, while suppressing the THC and CBN content. The identification and quantification of cannabinoid compounds were determined using UHPLC-MS/MS with external calibration. As a result, the RSM has shown good predictive capability with a *p*-value < 0.05, and the chosen parameters revealed to have a significant effect on the CBD, CBN and THC content. The optimal decarboxylation conditions for an extract richer in CBD than THC were set at 149.9 °C and 42 min as decarboxylation temperature and decarboxylation time, respectively. The extraction recoveries ranged between 96.56 and 103.42%, 95.22 and 99.95%, 99.62 and 99.81% for CBD, CBN and THC, respectively.

## 1. Introduction

Over recent decades, cannabis has been intensively studied due its therapeutic properties attributed to the presence of phytocannabinoids [1,2,3]. Δ^9^-Tetrahydocannabinol (THC) and cannabidiol (CBD) are the most studied of the 100 phytocannabinoids identified in the plant [4,5]. THC is largely responsible for psychoactive properties, and CBD exhibits anticancer, antiemetic and antiepileptic properties, among others [6,7,8]. These cannabinoids are originally produced from corresponding acidic analogues through decarboxylation via heat. They are released via decarboxylation under the influence of heat, light and oxygen [2,9]. Nowadays, several recovery approaches have been used to isolate cannabinoid compounds from its plant-based matrix. Extraction has played a vital role in the production of medicines; therefore, it is crucial to the development of cannabis-based medicinal products [10,11,12]. Conventional extraction techniques were found to have adverse toxic effects on the environment and human health [13,14,15]. Therefore, it is important to develop “green” extraction techniques for efficient extraction of the desired cannabinoids from the cannabis plant.

Most research has focused on the use of a supercritical carbon dioxide extraction technique (Supercritical CO_2_) as a green extraction method for the recovery of cannabinoids from the cannabis plant [5,16]. However, it requires a high capital investment, rendering the technique expensive. Pressurized hot water extraction (PHWE) has also been discovered to be a promising green technique for the extraction of phytochemicals from plant materials and a relatively cheaper alternative to supercritical CO_2_ [17,18,19,20,21,22].

Most important, the development of safe medicinal products from a plant, such as cannabis, requires selective extraction techniques that would eliminate the psychoactive effects of cannabis-based medicinal products. These techniques should produce CBD-rich extracts with low concentrations of the psychoactive constituents, THC and CBN. This has recently been achieved by Nuapia et al. [20] by the application of PHWE as an extraction technique selective to the desired CBD compound from cannabis seeds. In addition, the decarboxylation process is essential for maximizing the desired cannabinoid content in cannabis extract. This is normally carried out in an oven prior to extraction, and the heating temperature and time are considered to be the most important decarboxylation parameters [8,23,24]. In PHWE, the sample is placed in an extraction cell, which is heated in the oven at the desired temperature, before the dynamic extraction. This knowledge can be used to activate the cannabinoid compounds and perform selective extraction. However, the decarboxylation approach has not been incorporated in PHWE. Therefore, the current study focused on the optimization of in situ decarboxylation-PHWE conditions using response surface methodology to maximize the recovery of CBD and minimize the extraction of the psychoactive compounds, such as THC and CBN.

## 2. Materials and Methods

### 2.1. Chemicals and Reagents

All the chemicals used in this investigation were of analytical grade. Ethanol and methanol were employed as solvents and were purchased from Merck (Johannesburg, South Africa).

### 2.2. Plant Material

Cannabis seeds were collected from a farm in Johannesburg. Prior to processing, seeds were dried at 30 °C for 4 h, and the water content was found to be 3.5–5.1% in weight. The dried samples were crushed to powder and kept in a sealed container until further extraction.

### 2.3. Response Surface Methodology

Statistical techniques used to perform an optimization of experiments are essential in any method development applied in analytical processes, as they assess the interaction between factors that strongly influence the analytical methods. In the present investigation, a central composite design with two independent variables, namely, decarboxylation temperature (80–150 °C) and decarboxylation time (5–60 min), was used to assess the interaction between the two parameters in decarboxylation-PHWE of cannabinoid compounds. The independent parameters and their respective levels have been chosen in accordance with the studies reported by Moreno et al. and Wang et al. [5,9]. In agreement with central composite design, 12 experimental runs were performed to PHWE of cannabinoid compounds after a decarboxylation process. MODDE Pro (Sartorius Stedim Biotech, Malmö, Sweden) was used as statistical software. Each run represented a combination of parameter levels. For each experimental run, the amounts of THC, CBN and CBD were quantified, as summarized in Table 1. The output dataset from the central composite design was assessed by a multiple regression analysis model.

For each output, ANOVA analysis was used to investigate the significant terms in the model, and the outcomes are presented in Table 2. The F-test was applied to assess the significance of the coefficients of regression. The model adequacies were validated through R^2^, adjusted-R^2^ and prediction error sum of squares (PRESS). The response surfaces were generated through the regression coefficients. Coefficient plots and contour plots were generated to investigate the influence of the factors on each response.

### 2.4. Model Fitting and Accuracy

The response surface modeling was tested through the absolute average deviation (AAD), root-mean-square error (RMSE), mean absolute error (MAE), standard error of prediction (SEP), model predictive error (MPE) and chi-square statistic (*χ^2^*). Correlation coefficients (R^2^) were used to investigate the fit and accuracy of the model. The equations applied to compute these parameters are summarized in Table 3. The model abilities were also assessed by plotting the predicted values of RSM against the corresponding experimental values.

### 2.5. Pressurized Hot Water Extraction

Extraction was performed through a pressurized hot water extraction instrument built in house and published by Nuapia et al. [20]. It was equipped with a pump, water reservoir, GC oven, extraction cell and collector vessel. For each run, a mass of 5.00 g of cannabis material was transferred into an extraction cell. The extraction cell was preheated in the GC oven at various temperature and time conditions, as presented in Table 1. The purpose was to determine the best decarboxylation conditions which minimize the recovery of THC and CBN and maximize the extraction of CBD. Thereafter, the extraction of cannabinoids from decarboxylated cannabis powder was carried out for 30 min at 100 °C using the proposed PHWE set up.

### 2.6. Preparation of Standard Solutions and Calibration Curve

A 1000 ng mL^−1^ standard solution of CBD, CBN and THC was prepared by diluting a solution of 1 mg mL^−1^ of CBD, CBN and THC in methanol. The quantification of the PHWE extracts by UHPLC-MS/MS was completed with external calibration. The standards target compounds at concentrations ranging 0.5, 1, 5, 10, 20, 30, 50,100, 500 and 1000 ng mL^−1^ in methanol. An internal standard was not used in the analysis process.

### 2.7. UHPLC-MS/MS Conditions

The UHPLC-MS/MS system consisted of a ThermoFisher UltiMate 3000 HPLC and UHPLC Systems, and a dual pump was used. Chromatographic separation was performed on a C_18_ Wate column (4.6 × 100 mm, 3.5 μm particle size, Agilent 959961-902, (Bruker, Johannesburg, South Africa) with a guard column using a gradient mobile phase of water (0.1% formic acid): acetonitrile (0.1% formic acid) at a flow rate of 0.3 mL min^−1^ for 15 min. The column temperature was set at 30 °C, the autosampler temperature was maintained at 4 °C and the injection volume was 10 μL. Mass spectrometry analysis was performed as described by [9] Wang et al. 2016. Bruker-Compact-Qqtof-MS/MS equipped with an electrospray ionization (ESI) (Bruker, Johannesburg, South Africa) was used in positive ion-multiple reaction monitoring (MRM) mode for quantitative analysis. Precise MRM transitions were scrutinized for CBD, CBN and THC for maximum selectivity and sensitivity.

Partial method validation was conducted to assess the reliability and efficacy of UHPLC-MS/MS as an analytical technique for qualitative and quantitative determination of cannabinoids in the extract. The linearity, limit of detection (LOD), limit of quantification (LOQ), precision, accuracy and recovery were evaluated. Linearity was tested by evaluating the coefficient correlation (R^2^) for each calibration curve. The LOD and LOQ values for each analyte were calculated as ratios of standard deviation in response to the slopes of the calibration curves.

The extraction was performed in triplicate. Extraction recovery was calculated by comparing the peak area of blank matrix extract spiked with standards before and after the extraction procedure [9].

### 2.8. Quality Control Sample Preparation

For quality control, samples of *Moringa oleifera* leaves (used as a blank matrix) were spiked with concentrations of 0.5, 1, 10, 50, 100 and 500 ng mL^−1^ of standard.

## 3. Results and Discussion

### 3.1. Method Validation of UHPLC-MS/MS

Method validation was conducted to assess the reliability and efficacy of UHPLC-MS/MS as an analytical technique for qualitative and quantitative determination of cannabinoids in the extract. Validation of the PHWE extraction technique was also performed. For this, linear calibration curves were constructed for CBD, THC and CBN. The peak areas were plotted against the concentration of analytes over a range of 0.5, 1, 5, 10, 20, 30, 50, 100, 500 and 1000 ng mL^−1^. Mass spectrometry parameter settings were optimized in order to achieve the highest sensitivity, accuracy and resolution of the selected cannabinoid compounds (Table S1). A chromatogram of 10 ppm standard mixture of cannabinoid compounds and the PHWE extract at optimum conditions is presented in Figures S1 and S2, respectively. Supporting information on the fragmentation patterns obtained from the MRM mode is shown in Figure S3. 

In addition, the accuracy and precision of UHPLC-MS/MS were evaluated by using quality control samples of spiked Moringa powder, as were the recoveries achieved by PHWE extraction technique. Table 4 summarizes the linearity, correlation coefficient (R^2^), limit of detection (LOD) and limit of quantification (LOQ) estimated for each analyte.

The linearity of the curve is described by the R^2^ value, which was greater than 0.997 for all cases. The LOD and LOQ values estimated ranged between 0.03 ng mL^−1^ and 0.20 ng mL^−1^ and between 0.09 ng mL^−1^ and 0.61 ng mL^−1^, thus indicating good sensitivity.

Quality control samples were prepared by spiking 5.00 g of *Moringa oleifera* samples with standards (0.5–500 ng mL^−1^) of each the cannabinoids, and the samples were analyzed in triplicate. The accuracies were determined as the percentage of the mean of the measured concentrations to the nominal concentrations. The precisions were calculated as coefficients of variance (RSD%) among the three measured concentrations at each true concentration. The accuracies and precisions were calculated to satisfy the requirements that the mean of the measured concentrations was within 15% of the true concentration and that the RSD must fall within 15% [9]. These precisions and accuracies are reported in Table 5.

The accuracies were 99.31–100.55%, 84.07–100.40% and 96.52–101.50%, and the precisions were 1.34–11.43%, 1.72–7.28% and 2.25–5.68% for CBD, CBN and THC, respectively. Both the accuracies and precisions were within the acceptable range for all the analytes. Therefore, UHPLC-MS/MS quantifies the target analytes reliably.

Recovery was evaluated as well using Moringa oleifera powder spiked with standard concentrations of CBD, CBN and THC (0.5 ng mL^−1^ to 500 ng mL^−1^). The mean recoveries for each level were calculated by comparing the peak areas of each cannabinoid after the blank matrix was spiked with the standard concentrations, and then treated according to the extraction procedure with the peak areas before extraction procedure. The calculated extraction recoveries are shown in Table 6. In summary, the extraction recoveries ranged between 96.56 and 103.42%, 95.22 and 99.95% and 99.62 and 99.81% for CBD, CBN and THC, respectively. The observed recoveries were satisfactory, which demonstrated a highly efficient extraction procedure.

### 3.2. Response Surface Methodology

The response surface methodology was applied with the objective to optimize the factors affecting decarboxylation in the in situ decarboxylation-PHWE system. This was done in order to develop the best performing system which obtains CBD-rich extracts. The experiments were performed based on the experiment design defined by CCD. These experiments were carried out at all possible level combinations of temperature and time, and the response was given as the CBD, THC and CBN content. These results are presented in Table 2. Thereafter, the regression models were obtained by fitting the second-order polynomial equation to the experimental dataset. The adequacy of the regression models obtained was investigated using the model summary statistics or analysis of variance (ANOVA). The results are reported in Table 4. The goodness of fit of the established models was assessed by the R^2^. For all the models, the R^2^ value was close to unity, suggesting that the predicted model fits the experimental data well. The significance of each term on the regression model was assessed by considering the Fischer’s F-test values and probability *p*-values. In general, high F-values (F > 1) and low *p*-values (*p*-value < 0.05) indicate that the terms have significant influence on the regression models. From Table 2, it is clear that terms of the regression models for CBD, CBN and THC are highly significant since most of the terms have *p*-values < 0.05 and F-values > 1. Therefore, it was inferred that the independent factors (temperature and time) had a significant effect on the CBD, CBN and THC content.

The second-order polynomial model fitted to the data resulted in model Equations (1)–(3), which describe the response Y_x_ as a function of decarboxylation temperature (X_1_) and time (X_2_).

Y_CBD_ = 2.34 + 2.07X_1_ + 1.44X_2_ + 0.242X_1_^2^ − 0.026X_2_^2^ + 1.31X_1_X_2_ (1)

Y_CBN_ = 0.076 − 0.058X_1_ + 0.157X_2_ − 0.0352X_1_^2^ + 0.147X_2_^2^ − 0.037X_1_X_2_ (2)

Y_THC_ = 2.66 − 0.251X_1_ + 0.459X_2_ − 1.394X_1_^2^ + 0.520X_2_^2^ − 0.572X_1_X_2_ (3)

### 3.3. Model Fitting and Accuracy

The predictive efficiency of RSM was assessed based on the deviation of the predicted values from the actual CBD, CBN and THC content measured in the cannabis extracts. This was performed by calculating the statistical error parameters, namely, ADD (%), RSME, MAE, SEP (%), MPE (%) and chi-square (*χ^2^*). The results of the parameters are reported in Table 7.

**Figure 1:**
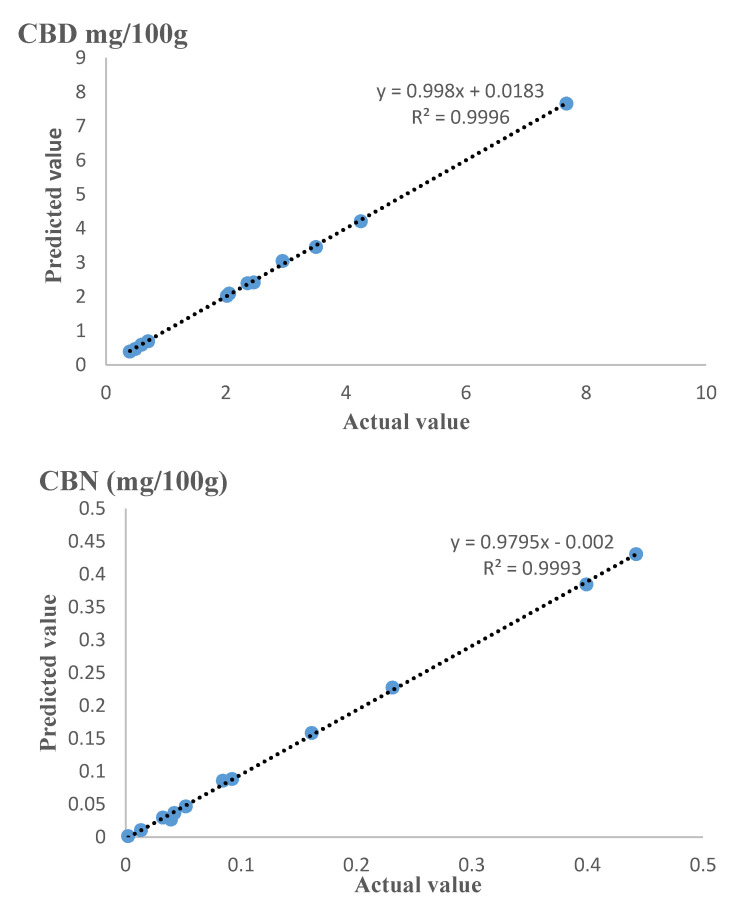
Predicted vs. actual values of cannabinoid responses.

The results demonstrate that the RSM has good predictive capabilities to determine the amount of cannabinoid analytes extracted using the in situ decarboxylation-PHWE system at experimental conditions. This was indicated by the low values of the statistical errors. Moreover, the plots comparing the predicted and actual values are presented in Figure 1.

Data fitting demonstrated that all data points were well concentrated around the selected unity-slope line for all three outputs. From this, it was inferred that the predicted and actual responses determined experimentally were in proximity, further attesting that the established model was valid, reliable and adequate to predict the CBD, CBN and THC contents accurately within the experimental region.

### 3.4. The Effect of Decarboxylation Conditions on the Extracted Amount of Cannabinoids

**Figure 2:**
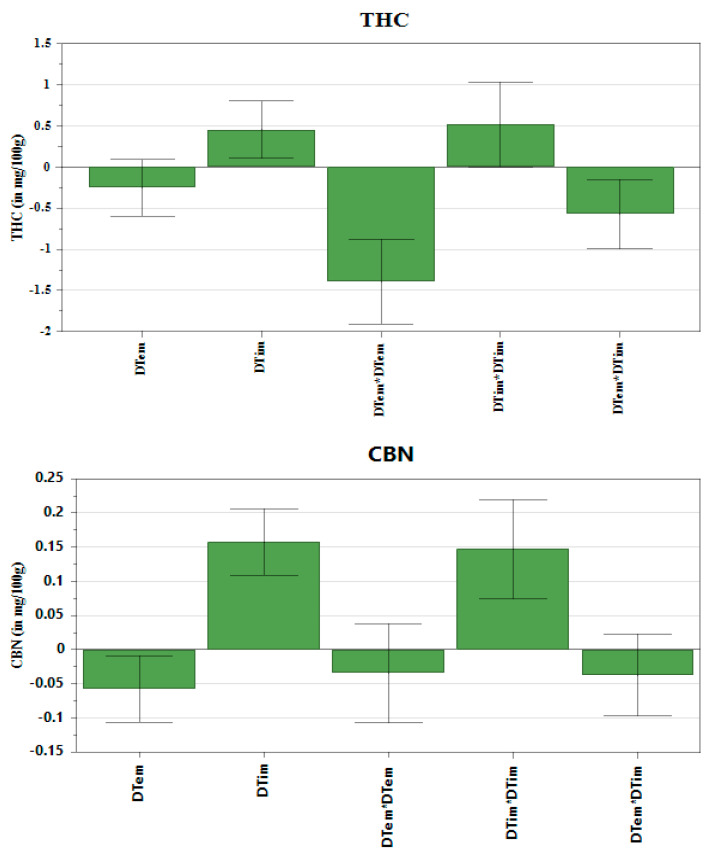

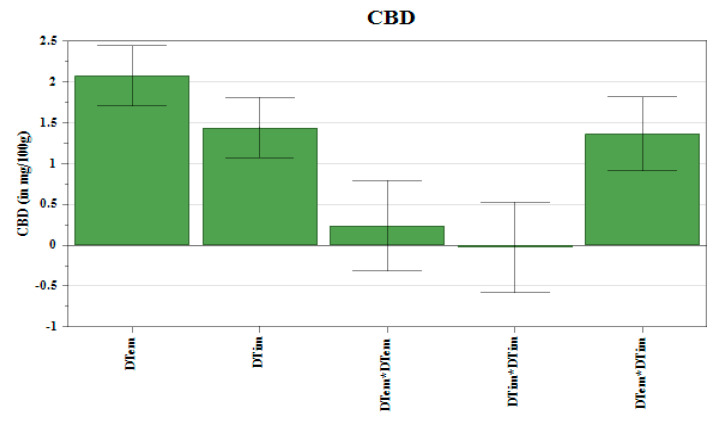
Coefficient plots.

**Figure 3:**
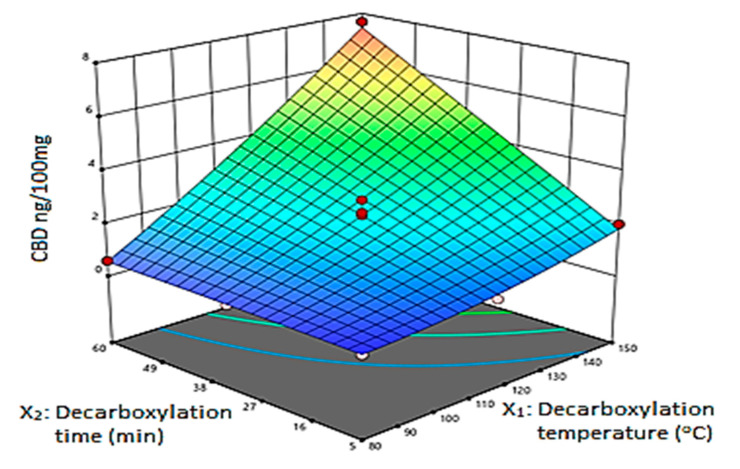

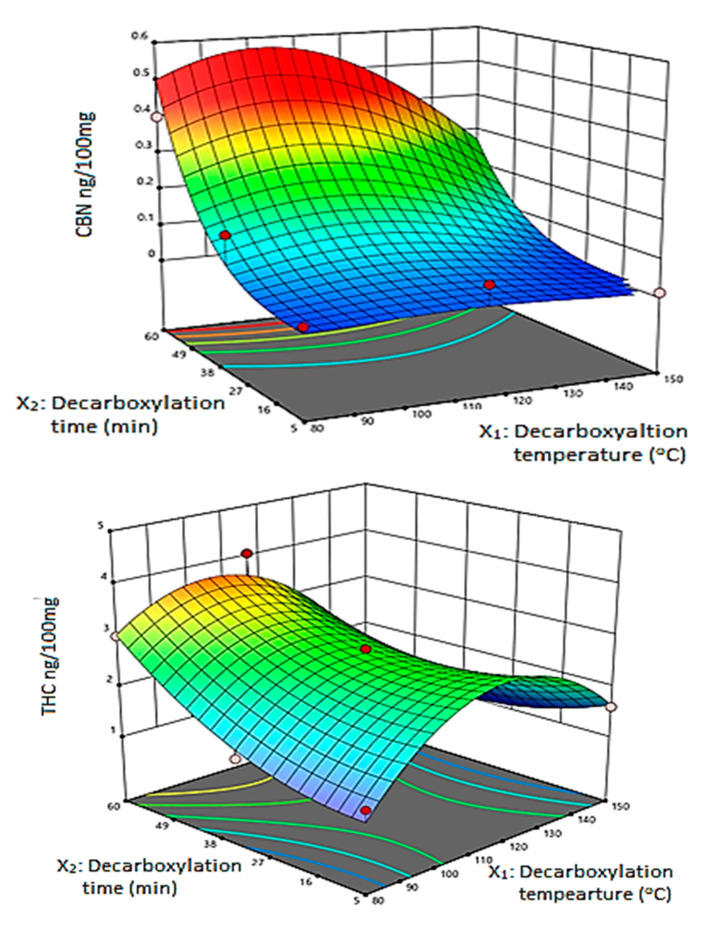
3D response surface plot.

The multilinear regression (MLR) was fitted to the data to construct coefficient plots (Figure 2) and the 3D response surface plots (Figure 3). The coefficient plots depict the significance of the terms on the THC, CBN and CBD content. The 3D plots illustrate the interactive effect of decarboxylation temperature and decarboxylation time on the amount of CBD, CBN and THC extracted. Regarding THC and CBN, the coefficient plots revealed that temperature had a significant, negative influence on the response. In contrast to THC and CBN, the decarboxylation temperature had a significant, positive influence on the CBD content. The decarboxylation time showed a positive, significant influence on the amount of CBD, CBN and THC recovered. Regarding the CBD content, factors showed that there was a substantial variation of the amount of CBD recovered when there was an interaction between temperature and time. Therefore, a steep rise in CBD yield was observed when both temperature and time were increased. The use of heat accelerates THC degradation, which results in CBN generation. Consequently, heating the *C. sativa* plant in the dark suppressed production of CBN, which resulted in low recovery. Wang et al. [9] made a similar observation that decarboxylation carried out in a dark vacuum oven suppressed CBN formation. The influence of temperature and time on the THC content was captured by the saddle-shaped response surface (Figure 3). The coefficient plots (Figure 2) show that both time and temperature had a significant influence on the amount of THC in the extract. The minimum yield was observed when the cannabis material was exposed to low temperatures over a short period of time.

A steady increase occurred when both temperature and time were increased, and the maximum THC content for the considered experimental region was observed at 60 min and approximately 110 °C. Above 125 °C, a loss of THC was observed due to degradation. However, the maximum yield of CBD was obtained at 150 °C after heating for 60 min. There was no decrease in CBD observed with rising decarboxylation temperatures at approximately 150 °C and time over the studied experimental range. According to Moreno et al. [5], the loss of CBD is expected to occur only when heating for 60 min at 160 °C and above.

### 3.5. Optimal Extraction Conditions

Desirable experimental conditions for in situ decarboxylation are needed to obtain CBD-rich cannabis extracts with low content of the psychoactive components, such as THC and CBN. An optimization tool in RSM was used to find the appropriate settings of decarboxylation temperature and time for high CBD recovery. The optimal conditions for decarboxylation temperature and time were found to be 149.9 °C and 42.2 min, respectively. The composition of the cannabis extract predicted using the established model is reported in Table 8.

For verification, extractions were carried out under the predicted, optimal conditions in triplicate. It was observed that the predicted amounts fell within the standard deviation of the experimental recoveries, therefore suggesting that the observed and experimental responses were in close agreement and that the established model was reliable for estimations and can be used for future predictions.

## 4. Conclusions

The UHPLC-MS/MS technique was validated for qualitative and quantitative analysis of cannabinoids in *C. sativa*. The performance of the analysis technique was investigated in terms of linearity, detection and quantification limits, accuracy, precision and recovery. Overall, it conveyed excellent analytical performance and was successfully applied as an analytical tool for efficient detection and quantification of cannabinoids. The in situ decarboxylation-PHWE system was optimized to produce cannabis extracts with high CBD content, while suppressing the THC and CBN content. Response surface methodology was carried out as an optimization tool. The established response surface model was used to investigate the influence of the decarboxylation temperature and time on the extraction yield of the cannabinoids. The model revealed the best conditions for the recovery of CBD rich extracts. The adequacy of the model was evaluated by error statistical techniques, including ADD, RSME, MAE, chi-square and R^2^_._ The results have shown that RSM offers excellent prediction and estimation capabilities. The optimal conditions predicted by RSM were 149.9 °C and 42.2 min for heating temperature and time, respectively. These experimental conditions helped to achieve the desired composition of the final extract with higher amounts of CBD than THC and CBN.

## Figures and Tables

**Table 1:** Design of experiment by response surface methodology.

	Factors		THC		CBN		CBD	
Run Order	Decarboxylatio Temperature	Decarboxylation Time	Actual Value	Predicted Value	Actual Value	Predicted Value	Actual Value	Predicted Value
8	80	5	1.239	1.036	0.032	0.03	0.395	0.404
5	115	5	2.529	2.547	0.084	0.086	0.696	0.703
9	150	5	1.597	1.469	0.013	0.011	2.012	2.031
3	80	32.5	1.347	1.368	0.161	0.159	0.486	0.479
12	115	32.5	2.749	2.628	0.052	0.047	2.458	2.427
6	150	32.5	1.409	1.357	0.002	0.002	4.246	4.219
4	80	60	2.997	3.061	0.399	0.385	0.589	0.601
11	115	60	4.055	3.987	0.442	0.431	3.499	3.468
10	150	60	1.066	0.958	0.231	0.228	7.672	7.672
7	115	32.5	2.749	2.538	0.092	0.089	2.052	2.098
1	115	32.5	2.549	2.348	0.039	0.027	2.355	2.401
2	115	32.5	2.349	2.245	0.042	0.037	2.939	3.056

**Table 2:** Analysis of variance (ANOVA) of the regression model for the recovery of cannabinoid compounds.

		THC			CBN			CBD		
	Df	CE	F-Value	*p*-Value	CE	F-Value	*p*-Value	CE	F-Value	*p*-Value
Model	5	0.1824	12.15	0.0142	0.0524	549.89	0.0364	0.0524	67.87	0.0001
a	1	0.1520	3.57	0.1085	0.0047	143.32	0.057	0.0047	190.88	0.0001
B	1	0.1673	5.70	0.0819	0.0082	1389.17	0.0185	0.0082	92.14	0.0001
aB	1	0.2144	10.78	0.0168	0.0054	49.92	0.0948	0.0054	55.1	0.0003
a^2^	1	0.2514	43	0.0010	0.0046	755.29	0.0264	0.0046	1.15	0.3239
B^2^	1	0.2458	3.48	0.1253	0.0098	91.10	0.0706	0.0098	0.0136	0.9111
PRESS		2.56			2.12			3.12		
R^2^		0.9428			0.9993			0.9826		
Adj R^2^		0.8428			0.998			0.9522		
Pred		0.9052			0.8956			0.9156		

**Table 3:** Equations of statistical errors.

Error	Equation
Absolute average deviation (ADD)	$\frac{\sum^{}(|Y_{o}-Y_{p}|/Y_{o})}{N}$
Root mean square error (RSME)	$\sqrt{\frac{\sum^{}{\left(Y_{o}-Y_{p}\right)}^{2}}{N}}$
Mean absolute error (MAE)	$\frac{\sum^{}|Y_{o}-Y_{p}|}{N}$
Standard error of predictions (SEP)%	$\frac{RSME}{Y_{o}}\times 100$
Model predictive error (MPE)%	$\frac{100}{N}\;\sum^{}\left|\frac{Y_{o}-Y_{p}}{Y_{p}}\right|$
Chi-square ($\chi^{2}$)	$\sum^{}\frac{{\left(Y_{p}-Y_{e}\right)}^{2}}{Y_{p}}$

where o is observed/actual response and *p* is predicted response.

**Table 4:** Linear calibration curves for determination of linear range, correlation coefficient, LOD and LOQ.

Analyte	Quantification Range (ng mL^−1^)	Fitting Equation	Correlation Coefficient (R^2^)	LOD (ng mL^−1^)	LOQ (ng mL^−1^)
CBD	0.5–1000	87.629x + 508.07	0.9971	0.199929	0.605847
CBN	0.5–1000	164.94x + 118.91	0.9999	0.031007	0.093959
THC	0.5–1000	93.594x + 416.33	0.9998	0.057157	0.173204

**Table 5:** Precision and accuracy of the determination of cannabinoids in *Moringa oleifera* powder (*n* = 3, mean ± SD).

Analyte	Concentration (ng mL^−1^)	Measured Concentration (ng mL^−1^)	Accuracy (%)	RSD (%)
	0.5	0.49 ± 0.02	98.00	5.40
CBD	1	1.00 ± 0.03	99.67	5.71
	10	9.50 ± 0.63	95.03	11.43
	50	49.69 ± 0.86	99.38	2.99
	100	100.55 ± 1.40	100.55	2.41
	500	496.55 ± 3.83	99.31	1.34
	0.5	0.46 ± 0.02	91.60	5.78
CBN	1	0.84 ± 0.03	84.07	6.76
	10	9.28 ± 0.39	92.80	7.28
	50	47.83 ± 0.86	95.66	3.11
	100	100.40 ± 3.70	100.40	6.38
	500	493.99 ± 4.90	98.80	1.72
	0.5	0.49 ± 0.01	97.33	5.17
THC	1	1.02 ± 0.03	101.50	5.68
	10	9.53 ± 0.20	95.30	3.68
	50	49.80 ± 0.72	99.61	2.50
	100	96.52 ± 2.06	96.52	3.69
	500	491.76 ± 6.38	98.35	2.25

**Table 6:** Recovery of cannabinoids from Moringa oleifera (*n* = 3) (mean ± SEM).

	0.5 ng/mL	1 ng/mL	10 ng/mL	50 ng/mL	100 ng/mL	500 ng/mL
CBD	96.56 ± 10.60	97.54 ± 5.65	103.01 ± 20.22	99.33 ± 2.98	103.42 ± 6.22	100.25 ± 3.36
CBN	95.22 ± 12.16	96.52 ± 6.63	99.92 ± 6.50	99.70 ± 4.71	99.71 ± 1.66	99.95 ± 1.50
THC	99.62 ± 10.58	98.82 ± 0.94	98.21 ± 1.47	99.81 ± 2.52	99.53 ± 1.38	99.30 ± 2.48

**Table 7:** Statistical errors determined for each response.

	ADD	RMSE	MAE	SEP%	MPE%	$\chi^{2}$
THC	5.724521	0.126433	0.10825	7.184042	5.724521	0.113183
CBN	7.342018	0.006739	0.005083	11.16061	8.991944	0.00804
CBD	1.555437	0.006739	0.029333	1.843651	1.530427	0.008005

**Table 8:** The experimental and predicted values for optimal in situ decarboxylation conditions.

Response	Criterion	Predicted Value mg/100 g	Experimental Value mg/100 g	Error (%)
THC	Minimize	1.047	1.030 ± 0.038	1.564
CBN	Minimize	0.051	0.493 ± 0.003	2.686
CBD	Maximize	5.717	5.780 ± 0.183	1.096

## Data Availability

The data presented in this study are openly available in referenced publications.

## Supplementary Materials

The following are available online.

## Sample Availability

Samples of the compounds are available from the authors.
