# Supplementary material for: In Situ Decarboxylation-Pressurized Hot Water Extraction for Selective Extraction of Cannabinoids from Cannabis sativa. Chemometric Approach
Source: Molecules. 2021 Jun 2;26(11):3343. doi: 10.3390/molecules26113343 (PMC8199533; doi:10.3390/molecules26113343)
Supplement: Supplementary file 1 [file molecules-26-03343-s001.zip › molecules-1209149-supplementary.pdf]

Table S1. multiple reaction monitoring (MRM) mode parameter settings

Compounds	Mass(amu)	Q1 m/z	Q3 m/z	Collision energy(V)
CBD	314	315.2383	193.1228	30
CBN	310	311.2017	223.1117	27
THC	314	315.2374	193.1236	30

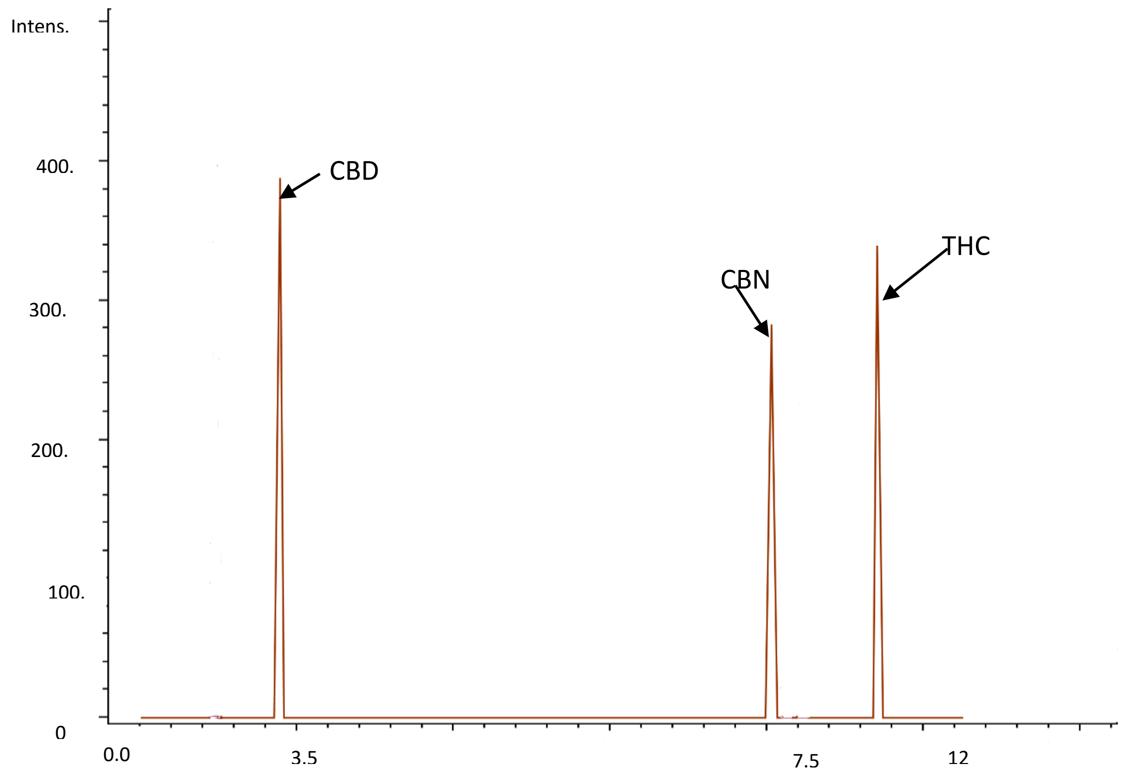

Figure S1 chromatogram of standard mixture of cannabinoid compounds

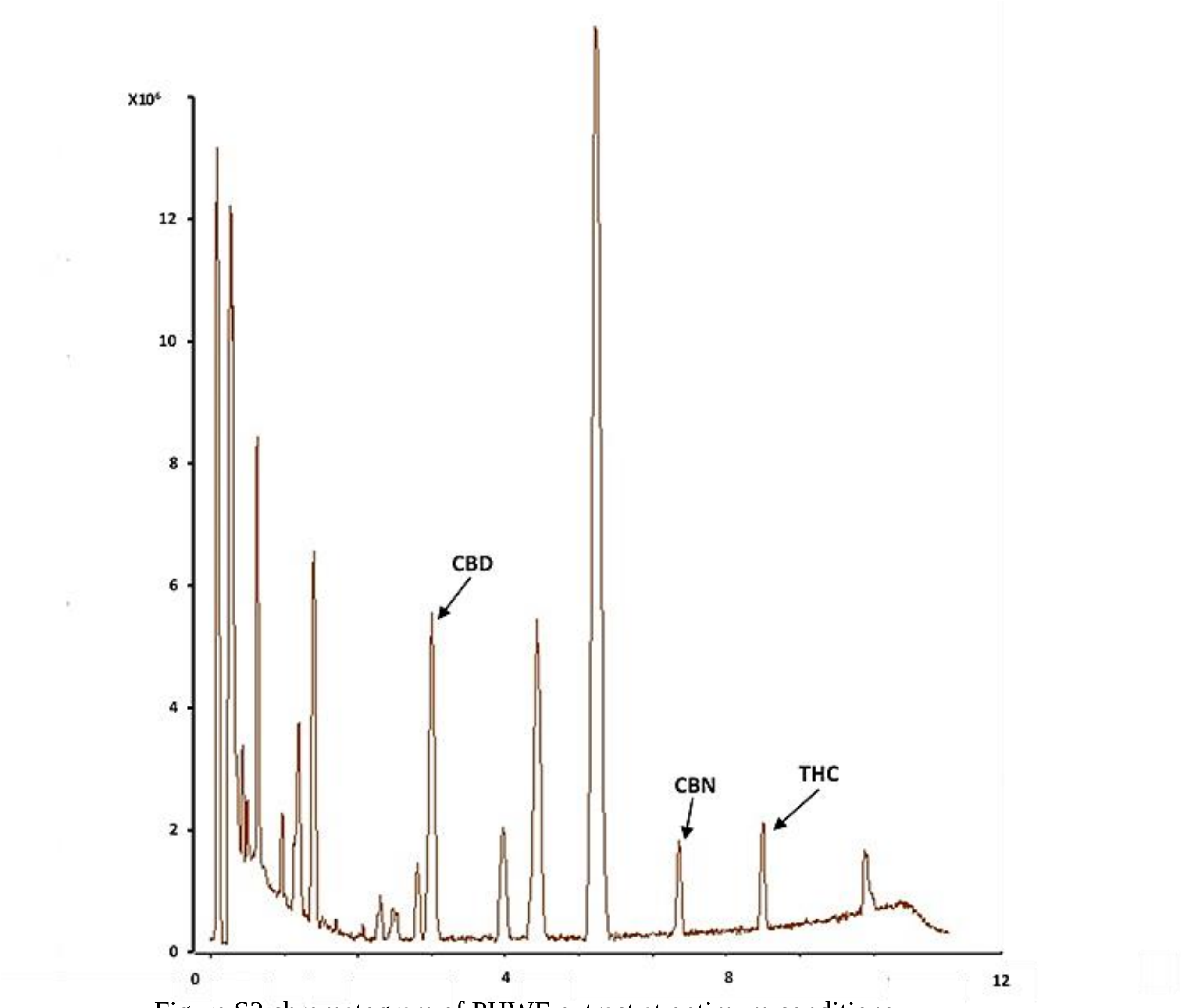

Figure S2 chromatogram of PHWE extract at optimum conditions

A

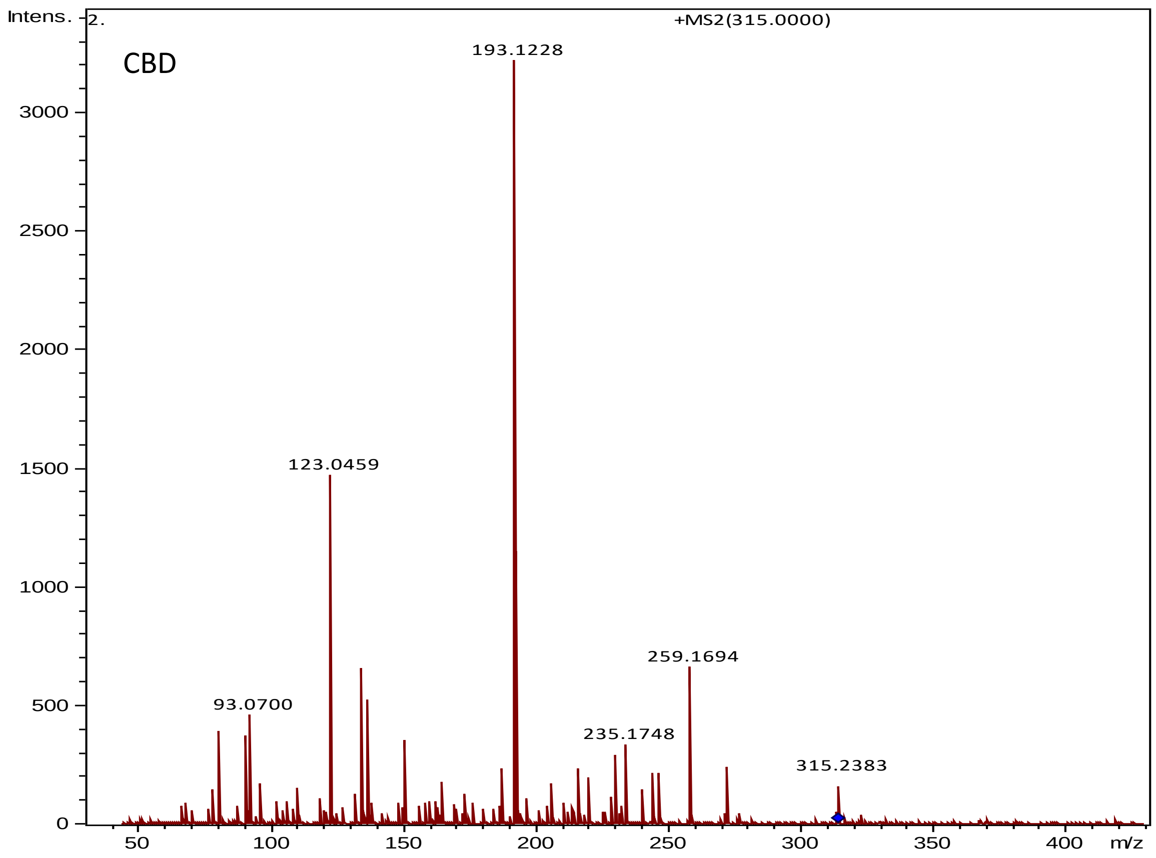
Intens.
2.
+MS2(315.0000)
CBD
193.1228
123.0459
93.0700
259.1694
235.1748
315.2383
3000
2500
2000
1500
1000
500
0
50
100
150
200
250
300
350
400
m/z

B

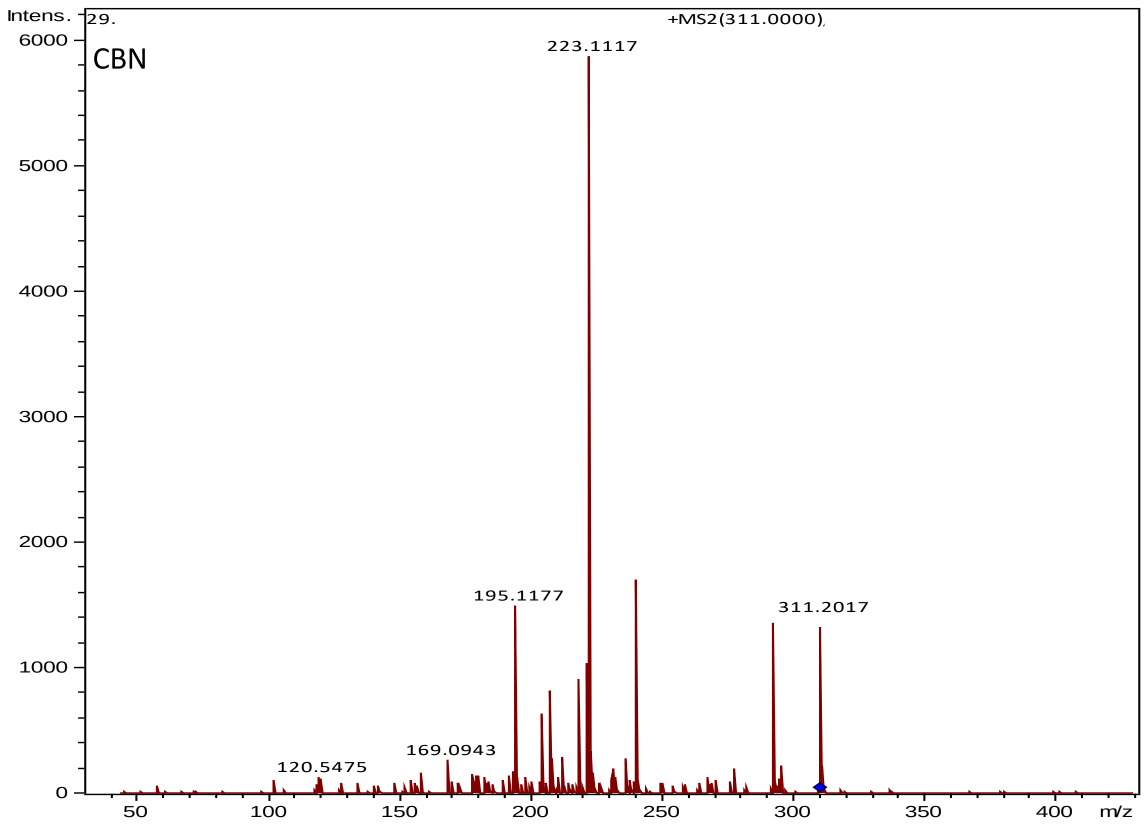
Intens.
29.
+MS2(311.0000)
CBN
223.1117
195.1177
311.2017
120.5475
169.0943
6000
5000
4000
3000
2000
1000
0
50
100
150
200
250
300
350
400
m/z

C

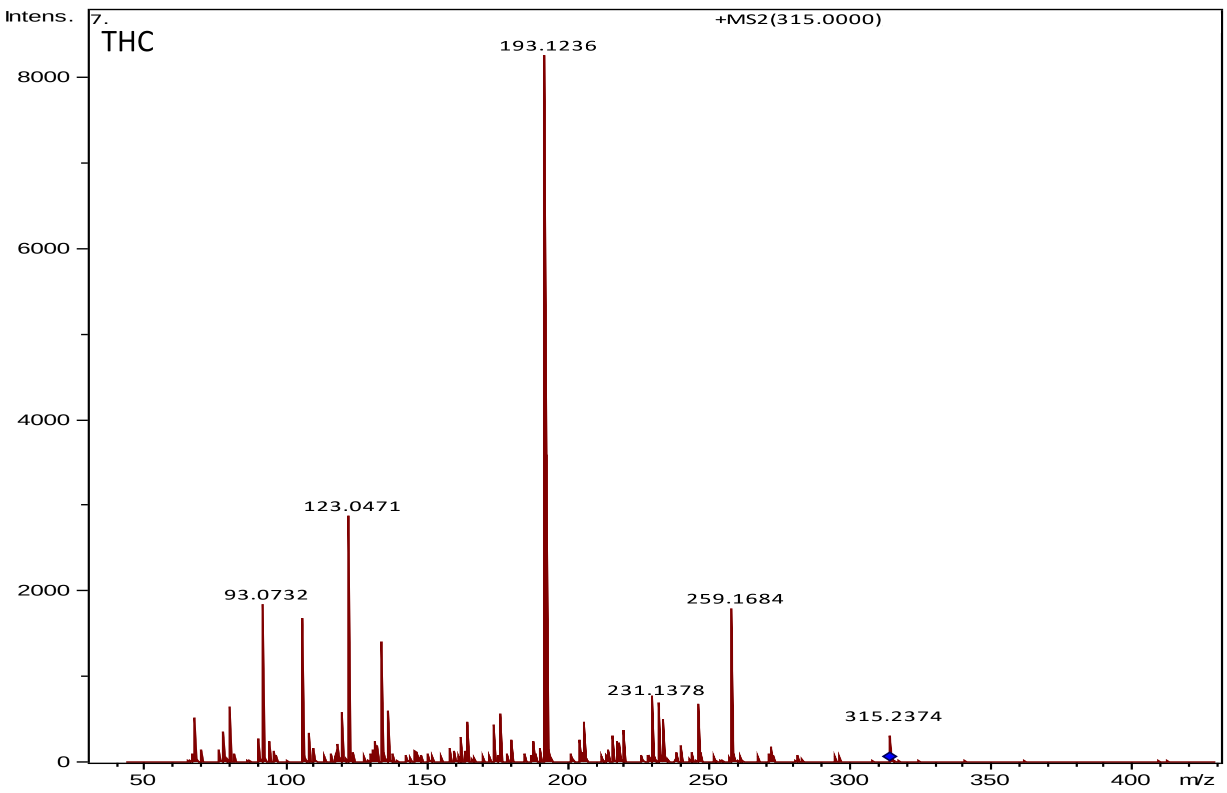

Figure S3 MS/MS fragmentation spectrums of cannabidiol compounds in positive: CBD (**A**), CBN (**B**), THC (**C**)
